# Southern ocean sea level anomaly in the sea ice-covered sector from multimission satellite observations

**DOI:** 10.1038/s41597-022-01166-z

**Published:** 2022-03-02

**Authors:** Matthis Auger, Pierre Prandi, Jean-Baptiste Sallée

**Affiliations:** 1 Sorbonne Université, CNRS, LOCEAN, Paris, France; 2 Collecte Localisation Satellite, Toulouse, France

**Keywords:** Physical oceanography, Ocean sciences, Techniques and instrumentation, Research data, Scientific data

## Abstract

Despite its central role in the global climate, the Southern Ocean circulation is still one of the least understood ocean circulation systems of the planet. One major constraint to our understanding of this region is the challenge of observing ocean circulation in the seasonally sea ice sector of the Southern Ocean. Here, we present a new Sea Level Anomaly (SLA) product, focusing on the subpolar Southern Ocean and including its sea ice covered parts from 2013 to 2019. Combining observations from multiple satellites, including Cryosat-2, Sentinel-3A, and SARAL/AltiKa, processed with state-of-the-art algorithms, allows an improvement in spatial and temporal resolution compared with previous products. Validation is made by comparing our estimate with existing SLA products, cross-comparing estimates from individual satellites in the sea ice zones, and comparing the time series of the product with a Bottom Pressure Recorder in the Drake Passage.

## Abstract (summary)

**Table:** 

Measurement(s)	Sea Level Anomaly
Technology Type(s)	Satellite Altimetry
Sample Characteristic - Location	Southern Ocean

## Background & Summary

The Southern Ocean is a central element of the climate system, yet it is very poorly observed, understood, and not well represented in climate models^1^. The Southern Ocean is the main anthropogenic heat and carbon sink of the world’s oceans^1,2^, and acts as a major hub distributing physical and biogeochemical properties around the globe^3,4^. Despite this importance, the Southern Ocean, and particularly its seasonally ice-covered subpolar region, remains poorly sampled, which impedes long-term monitoring of its change and limits progress in its representation in climate models^1,5^. In particular, very little is known about the drivers of ocean circulation in the subpolar seas and how they are affected by current global climate change^1,6^.

In this paper, we revisit the processing of satellite altimeter observations developed over the past decades to produce a new and unprecedented observational dataset of sea-level anomalies (SLA) and geostrophic velocity anomalies in the Southern Ocean subpolar seas from a multi-satellite approach. Since 1992, satellite altimetry has helped to map the global ocean geostrophic circulation through high precision sea level measurements while allowing a better understanding of the Earth’s climate variability and response to climate change^7^. The number of satellites sampling the ocean is now larger than ever, creating new possibilities in terms of combination and sea level mapping resolution. Daily and global multi-mission products such as the Data Unification and Altimeter Combination System^8^ (DUACS) reach a horizontal resolution of 100 km at high latitude^9^. However, these products do not include the ice-covered regions of the global oceans, even though conventional satellite altimetry can help to understand the open ocean parts of the polar oceans^10^. Dedicated processing needs to be used over ice-covered areas.

Since the early years of altimetry, many studies have been conducted to understand how to process and obtain valuable ocean observations in sea ice zones. Specular reflectors such as leads or calm open water polynyas were first detected in the altimeter footprint by using an airborne radar altimeter and comparing with large-format aerial photography^11^. Later, a first ocean / sea ice classification technique was developed using ERS-1 satellite altimeter along with a new threshold retracking algorithm for sea ice, taking into account the fact that conventional models were not able to retrack powerful specular sea ice echoes^12^. The first mean sea surface and sea surface height variability product in the ice-covered Arctic was released using ERS altimeters^13^. Using a very similar processing scheme but different satellites, various datasets such as sea ice thickness^14^, mean sea level trends^15^ and sea surface height studies^16–19^ were released in the Arctic and helped uncover its changes and variability. The first sea surface height variability maps in the subpolar Southern Ocean were limited to its ice-covered parts^17^. Based on Cryosat-2 observations from 2012 to 2016, the first monthly sea surface height product of the whole Southern Ocean was produced^20,21^, allowing to document the seasonal climatology of the subpolar sea surface height, interannual variability and forcings^22^. In the present study, we extend this effort by combining observations from multiple satellites, thereby allowing for higher spatial and temporal resolution than previously done. We also leverage recent radar altimetry signal processing advances: a neural network based waveform classification for lead detection and a physical retracker algorithm that alleviates the need for ad-hoc bias correction between the open ocean and sea ice sectors.

## Methods

### Data source

#### Satellite altimeters

We use observations from three satellite altimeters: Cryosat-2, Sentinel-3A, and SARAL/AltiKa, which we present below in turn (see also Table 1).

**Table 1:** Altimeters characteristics.

Altimeter	Launch Date	Mode	Sampling Frequency	Inclination
SARAL/AltiKa	2013/02	LRM	40Hz	99°
Cryosat-2 (sea ice)	2010/04	SAR	20Hz	92°
Sentinel - 3A	2016/02	SAR	20Hz	99°

Cryosat-2 is an ESA mission, which was launched in April 2010. Its SIRAL instrument is a Ku-band (i.e. frequency range from 13 to 17 GHz) radar altimeter working in three different modes: Low Resolution Mode (LRM) over most of the ocean, SARM (Synthetic Aperture Radar Mode) on the sea ice, and SARInM (Synthetic Aperture Interferometric Mode) on the temperate land ice (i.e. every land ice region except the Greenland and Antarctic ice caps) and the ice-sheet margins^23^. Only the ESA Cryosat-2 ICE SAR Baseline-C L1b dataset was used for this study. This dataset includes the sea ice zones within Cryosat-2 SARM mask. SARM allows a better along-track resolution via the use of the Delay Doppler processing, reaching about 400 m of effective resolution compared to the 8 km resolution of conventional altimeters^24^.

Sentinel-3A carries the dual-frequency Synthetic Aperture Radar Altimeter (SRAL) instrument, which was launched in 2016^25^. Only the Ku band is used for the altimetric measurements^26^. Sentinel 3A CNES Processing Protocol (S3PP) data are used as they include the Zero-Padding and Hamming processings, which are necessary for SAR data in sea ice zones^27^.

SARAL is a CNES-ISRO (Centre National d’Etudes Spatiales, Indian Space Research Organisation) satellite, which was launched in February 2013. It carries AltiKa, a conventional (LRM) Ka-band (i.e. 35.75 GHz) radar altimeter, which allows a smaller footprint than a Ku-band radar (4 km versus 15 km for an identical orbit) and a higher sampling frequency (40 Hz versus 20 Hz). The primary objective of this high-resolution ocean topography satellite is the observation of ocean mesoscale circulation^28^.

### Data processing

Combining multiple missions into a single product requires care to adequately take into account differences between instruments. In our study, we must take into account the difference between SARAL/AltiKa LRM altimeter, and Cryosat-2 and Sentinel-3 SAR altimeters. Low-resolution mode altimeters are historically considered as the conventional instruments. Their resolution is limited by the length and width of the pulse. SAR altimeters allow a better along-track resolution due to the multi-look of the target along the path of the satellite^27^. The resulting waveforms are narrower, and therefore dedicated processing is required. We present below the processing, that was organised in five main steps: (i) classification, (ii) retracking, (iii) geophysical corrections, (iv) bias correction, and (v) mapping.

#### Classification

**Fig. 1:**
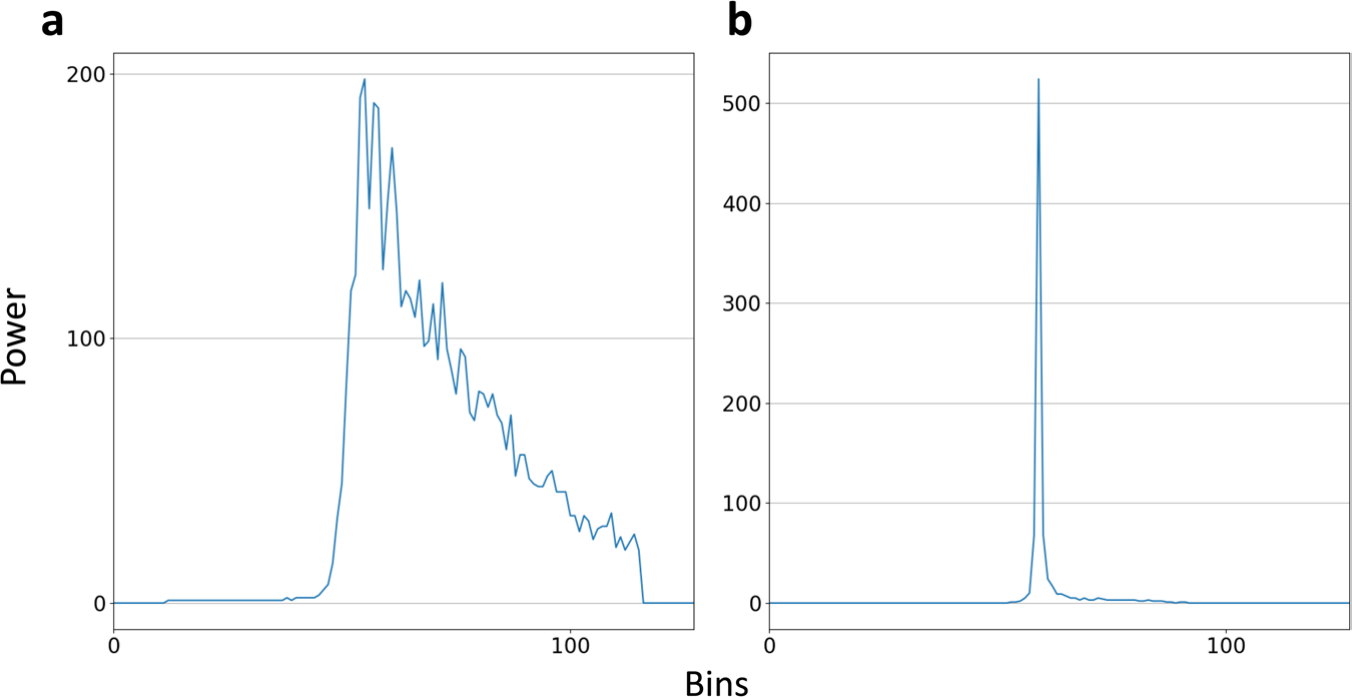
Examples of waveforms from SARAL/AltiKa altimeter (**a**) Brownian echo from open ocean. (**b**) Peaky (specular) echo from a lead.

The SLA field is built using open ocean and lead echoes. Data points located on the continent, continental ice, or sea ice are discarded. For that we use a neural network waveform classification algorithm^29^ validated using SAR images^30^. Each echo is affiliated one of 12 classes representing various waveform shapes and surface types. This classification is complemented with the traditional multiple criteria approach, considering backscattered power and pulse peakiness^13,16^. Open ocean and lead data are selected and processed separately. Radar returns are very different in terms of specularity and backscattering depending on the surface type and roughness. In the open ocean, the wind on the free surface creates a high surface roughness, leading to the reception of a Brownian type of waveform^27^ (Fig. 1a). In the sea ice areas, specular echoes (Fig. 1b) are mostly representative of reflection from the leads, their free surface being protected from the wind by the neighboring floes^13^. Thus, the waveform is peakier and more powerful than in the open ocean. We do not investigate for differences between melt ponds and leads in the classification, as they are mostly specific to Arctic sea ice surface melt in summer, and less of an issue in the Antarctic^31^.

#### Retracking

Waveforms represent the power backscattered from multiple facets over the surface in the altimeter footprint, which are located at different ranges from the altimeter. The retracking process allows the retrieval of the geophysical parameters from these waveforms^27^. Retrackers can be either physical or empirical. Physical retrackers, such as SAMOSA SAR for Sentinel-3A^32^, fit an analytical model to the waveform to estimate quantities such as epoch, Significant Wave Height (SWH), and radar backscatter. Physical retrackers are commonly used in the open ocean but most of them are not able to retrack specular waveforms from sea ice. Sea ice echoes are commonly retracked using empirical retrackers such as the TFMRA^33^ (Threshold First Maximum Retracker Algorithm). In this case, geophysical parameters are estimated by empirical criteria^12^.

Commonly-used physical retrackers are not able to process open water or specular waveforms in the same way^27^. In previous studies combining sea ice and open ocean sea-level observations^20,21^, Cryosat-2 L1b data were processed using a physical retracker in the open ocean and an empirical (TFMRA) retracker over sea ice, and the bias between the two retrackers was corrected empirically. In these studies, the bias between both zones was corrected by computing SLA differences along the sea ice margins, on grid points where it is possible to find both peaky and Brownian waveforms for each month, or at the transition from open ocean to sea ice along a satellite track. Such bias estimates are based on a limited number of measurements and are therefore highly uncertain. They can also create artifacts in the resulting sea-level anomaly product^34^ that are difficult to distinguish from genuine ocean variability. To try to alleviate this bias, here, we use a new retracker that has been developed for LRM altimeters, by modifying the conventional physical retracker for the Brownian echoes, making it flexible enough to retrack specular waveforms from the leads^29^. This ‘adaptive’ retracking for both open ocean and sea ice echoes was made possible by considering the variation of backscattering power with incidence angle, allowing a processing continuity between the two surfaces for the same altimeter. Other similar solutions have been recently developed for other satellites, such as CS2WfF^35^ or SAMOSA+^36^, and might be implemented in future versions of this dataset in the coming years.

The new retracker applies to open ocean and sea ice echoes consistently, allowing to retrieve consistent sea level anomaly maps without empirical bias correction at the sea ice edge, but it is currently only available for SARAL/AltiKa. For Cryosat-2 and Sentinel-3A in the sea ice zones, we process observations with the TFMRA retracker, but we reference Cryosat-2 and Sentinel-3A to the SARAL/AltiKa observations (see section "bias correction" below).

Another advantage of using a physical retracker in the leads is that the algorithm models the full waveform, allowing the consideration of residual winds for the retracking. In comparison, the TFMRA algorithm TFMRA does not consider the effect of the wind, potentially leaving part of the signal uncorrected.

#### Geophysical corrections

Geophysical corrections are listed in Table 2. Satellite orbit estimation is computed using POE-E algorithm^37^. Once the range is computed, geophysical corrections are applied to remove the tidal and atmospherical components of the range and to compute the sea surface height. The same corrections are used for each mission when possible for homogeneity. As in previous studies^20^, we do not apply the high frequency dynamic atmospheric correction in the sea ice zone under the assumption that the impact of the wind on the free surface in the leads is limited.

**Table 2:** Geophysical Corrections applied to each altimeters.

	SARAL/AltiKa	Cryosat-2	Sentinel-3A
Orbit	POE-E^37^	POE-E^37^	POE-E^37^
Ocean Tide	FES14^38^	FES14^38^	FES14^38^
Polar Tide	From Desai *et al*.^56^	From C2 Product	From Desai *et al*.^56^
Earth Tide	Elastic response to tidal potential (from Cartwright and Tayler, 1971)^57^	Elastic response to tidal potential (from Cartwright and Tayler, 1971)^57^	Elastic response to tidal potential (from Cartwright and Tayler, 1971)^57^
Dry Tropospheric Correction	Model from ECMWF gaussian grids	Model from ECMWF gaussian grids	Model from ECMWF gaussian grids
Wet Tropospheric Correction	Model from ECMWF gaussian grids	Model from ECMWF gaussian grids	Model from ECMWF gaussian grids
Ionospheric Correction	GIM^40^	GIM^40^	GIM^40^
Sea State Bias	Non-Parametric^58^	Non-Parametric^58^	Non-Parametric^58^
Dynamic Atmospheric Correction	MOG2D high frequencies (open ocean) and inverse barometer forced with atmospheric ECMWF pressure and wind field (Carrere and Lyard, 2003)^41^	MOG2D high frequencies (open ocean) and inverse barometer forced with atmospheric ECMWF pressure and wind field (Carrere and Lyard, 2003)^41^	MOG2D high frequencies (open ocean) and inverse barometer forced with atmospheric ECMWF pressure and wind field (Carrere and Lyard, 2003)^41^
Mean Sea Suface	CNESCLS15^43^	CNESCLS15^43^	CNESCLS15^43^

Ocean tide is corrected using FES2014 model^38^. Ocean tide errors are estimated by computing the standard deviation of the difference between one year of FES2014 and GOT4V10^39^ tide signal on a 1° grid covering the whole Southern Ocean. Errors obtained are of the order of 1 cm in the open ocean, 2 cm in the seasonally ice-covered ocean, and about 8 cm in the permanently ice-covered Southern Ocean. This error is partially corrected using the long-wavelengths correction (see section "Mapping", below). The Global Ionosphere Maps (GIM) ionospheric correction is applied^40^. Wet and dry tropospheric corrections, along with MOG2D high-frequency^41^ and inverse barometer low frequency dynamic atmospheric corrections are taken from ECMWF (European Centre for Medium-Range Weather Forecasts) operational model Gaussian grids (https://www.ecmwf.int/en/forecasts/dataset/operational-archive).

An objective analysis (OA) mapping method is used to convert along-track measurements (Level 2) into a gridded product (Level 4). The OA method requires that time-mean is removed from the data to be mapped^42^. Here, the time-mean that is removed from the along-track observations before mapping is the Mean Sea Surface (MSS) CNESCLS15, which is based on open ocean measurements. Seasonally ice-covered regions of the MSS represent therefore a mean state of the ice-free time of the year, and permanently ice-covered regions are extrapolated^43^. As an alternative method which would not use the MSS with potential errors in sea ice covered region, one could instead reference the observations to the geoid, and then grid them, but this alternative method would downgrade the final resolution product, because the geoid is not known at short scales (typically less than 100 km)^44^.

Editing is performed once the fully corrected SLA is estimated. It uses empirical thresholds based on sea ice concentration, peakiness and backscatter coefficient of the echoes to discard possible errors. The sea ice concentration data is obtained from EUMETSAT OSI-450 until the end of 2015, and OSI-430-b from 2016^45^. In the open ocean, the remaining outliers are removed using an iterative editing method. This method consists in applying a 124-points low-pass Lanczos filter on the along-track data, and removing outliers identified as each measurement such as $\left|SLA-SL{A}_{filtered}\right| > 3\ast std\left(SLA-SL{A}_{filtered}\right)$. This editing step is conducted multiple times until outliers represent less than 0.1% of the measurements. The iterative editing step is not performed in the sea ice regions, as the sampling is too irregular for applying such along-track filters.

#### Bias Correction

There are evidences that correcting a monthly bias between retrackers at the sea ice margins as in previously available SLA products^20,21^, does not properly correct for the retracking bias in the entire sea ice zone^34^. SARAL/AltiKa ‘adaptive’ retracking allows a continuous and consistent SLA computation in the open ocean and in the leads, without the need of a bias correction. We therefore use SARAL/AltiKa as our reference mission to properly correct bias between open ocean and sea ice sectors of other missions. Monthly SLA maps at a horizontal resolution of 1x1 degree are therefore constructed with SARAL/AltiKa observation only, as well as maps computed independently for Cryosat-2 lead observations, Sentinel-3A lead observations, and Sentinel-3A open ocean observations. Median biases between the SARAL/AltiKa map and every other map are computed and used to correct each mission and surface each month. This allows us to estimate inter-mission biases which are representative of all the data coverage of the mission and retracker, and not only at the sea ice margins.

#### Mapping

The combination of all along-track observations from each mission into a daily gridded dataset is done by adapting the latest DUACS-DT2018 mapping procedure^8^ to our region of interest. It is based on an optimal interpolation (OI)^46–49^. This OI method uses an *a priori* statistical knowledge on the covariance functions of the sea level anomalies^50^ and the data noise to compute the SLA at an estimation point^46^. Here, the estimation points are the gridpoint of the 25-km ease2 grid centered around the geographic South Pole and reaching 50°S. A selection of observations within a space-time subdomain around the estimation gridpoint and the date of interest is used for the interpolation. We therefore need to define a subdomain around each gridpoint, the expected variance of sea-level, and data noise.

**Fig. 2:**
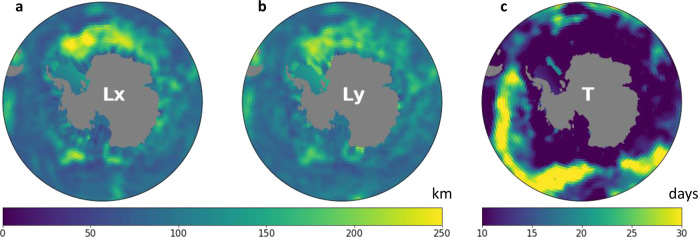
Correlation scales of Sea Level Anomaly computed on GLORYS12 model outputs. (**a**) Zonal correlation scales. (**b**) Meridional correlation scales. (**c**) Temporal correlation scales.

A subdomain is computed for every point of the grid, and its size depends on the correlation scales of the sea level anomaly at that given gridpoint. Only the input files are modified from the DUACS-DT2018^8^ mapping procedure. The correlation scales are computed from 2016 daily outputs of a global ocean model at 1/12° resolution, which assimilates observations (GLORYS12^51^; Fig. 2). Minimal temporal correlation scale has been set to 10 days, and the largest values reach 35 days in the most stable meanders of the Antarctic Circumpolar Current (ACC). Spatial correlation scales range from 150 to 300 km.

**Fig. 3:**
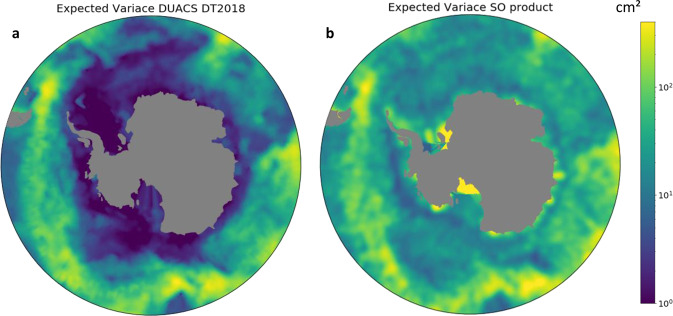
Expected variance input for the Optimal Interpolation method, (**a**) for DUACS-DT2018^8^ product, (**b**) recomputed for the Southern Ocean regional product.

The expected variance of the signal is investigated from the DUACS-DT2018 variance. We find however that in our region of interest, south of the ACC, DUACS-DT2018 has very low variance (Fig. 3a), much lower than SLA variance from Armitage *et al*.^20^. SLA product. Therefore, we here chose to compute the expected variance from our own observations rather than using DUACS-DT2018. We use a recursive method working on one arbitrary chosen year from March 2018 to March 2019. The recursive method starts by producing SLA maps for the given year, by using a large expected variance. We then compute the variance from this series of maps and recompute a series of maps, but now using the revised variance estimate. And we continue to repeat the procedure until the variance converges toward a stable map. This process converges at the fourth iteration, with a reduction of less than 3% of the variance between the last two iterations. This newly produced variance has the same order of amplitude as the one in DUACS DT2018 in the open ocean, but without the large drop in variance in seasonally ice-covered areas that was present in DUACS DT2018 (see Fig. 3b).

The expected noise is computed by adding MSS CNES-CLS15 error^43^ to the instrumental errors (same as applied in DUACS) with a factor that depends on the measurement frequency: the noise from data acquired at a frequency *freq* is $\sqrt{freq}$ times higher than when acquired at 1Hz. Thus, as the AltiKa instrument from the SARAL mission is sampling at 40Hz, the noise applied to this mission for the mapping will be $\sqrt{2}$ higher than Cryosat-2 and Sentinel-3A altimeters sampling at 20Hz.

We apply a long-wavelength error correction during the mapping to remove along-track correlated signals^46,47^. Such signals can arise from residual orbit, tide, or dynamic atmospheric correction errors and produce’stripes’ on SLA maps when not accounted for. This correction is done by modifying the error covariance for the along track measurements and evaluating the long-wavelength error of the along track data around the estimation point^46^.

An estimation is computed for each gridpoint every day, allowing a daily, 25 km resolution dataset in the Southern Ocean south of 50°S. Finally, once the product is corrected, we remove the temporal mean of the SLA for a better concordance and comparison with previously published products. Our SLA product represents therefore anomalies from the 2013-2019 mean sea level.

## Data Records

Dataset is publicly available on SEANOE^52^ with the 10.17882/81032. The Southern Ocean SLA and geostrophic currents product is distributed as a single NetCDF file *dt_antarctic_multimission* - *_sea_level_uv_20130401_20190731.nc*. It contains daily Sea Level Anomalies, associated geostrophic currents anomalies, and mapping formal error from April 2013 to July 2019. Individual fields are described in Table 3. All fields are mapped daily on a 25km EASE2 grid^53^ south of 50°S. Daily grids are dated using the number of days since 1950/01/01.

**Table 3:** Fields of the Sea Level Anomaly product *dt_antarctic_multimission_sea_level_uv_20130401_20190731.nc*.

Field	Description
longitude	Longitude (°)
latitude	Latitude (°)
time	Days since 1950/01/01
sla	Sea Level Anomaly (m)
U	Zonal Geostrophic Current Anomaly (m/s)
V	Meridional Geostrophic Current Anomaly (m/s)
formal_err	Formal Error (m)

## Technical Validation

### Validation

Validation is performed by comparing the mapping outputs with other data sources. Pearson correlation is used to compare time series. Associated Pearson p-value is used for the evaluation of the significance of the correlation. The local temporal correlation scale (from 10 to 30 days, Fig. 2) is defined as the interval between two independent measurements. Correlation significance is assessed at the 99% confidence level.

#### Concordance with DUACS DT2018 in the open ocean

The first validation of our new product is obtained for the open ocean region, where we compare our results to the daily DUACS DT2018 product^8^, which has been extensively used and validated in various regions of the global open ocean. Between 2014 and 2018, 85% of total ice-free grid points (grid points that never reach a 1% SIC within the 4 years) have a significant correlation with the DUACS product greater than 0.80. The remaining discrepancies can come from differences in the mapping parameters as well as in the sampling, as the number of satellites used in each product is different.

#### In-situ validation

Validation in the sea ice zones is limited by the poor number of *in situ* time series relevant for sea-level anomaly validation in the Southern Ocean. For instance, tide gauges are very sparse along the Antarctic coast, and their sampling period is often not overlapping with our time series. Most of the Permanent Service for Mean Sea Level (PSMSL) dataset ends before 2013 (for instance at Casey or Cape Roberts tide gauge). For the few stations where there is a time overlap with our product, their coastal and landlocked locations (for instance, Scott Base, Rothera, Argentine islands) make comparisons extremely difficult with satellite altimetry. There would be a strong need for coastal tide gauges in regions more representative of the open ocean (i.e. less landlocked) around Antarctica, corrected for tides and atmospheric pressure, in order to robustly validate future subpolar Southern Ocean products. Here, we nevertheless attempt to validate using a bottom pressure recorder (BPR) time-series in the open ocean, and second by comparing sea-level anomaly maps produced independently by single altimeters.

**Fig. 4:**
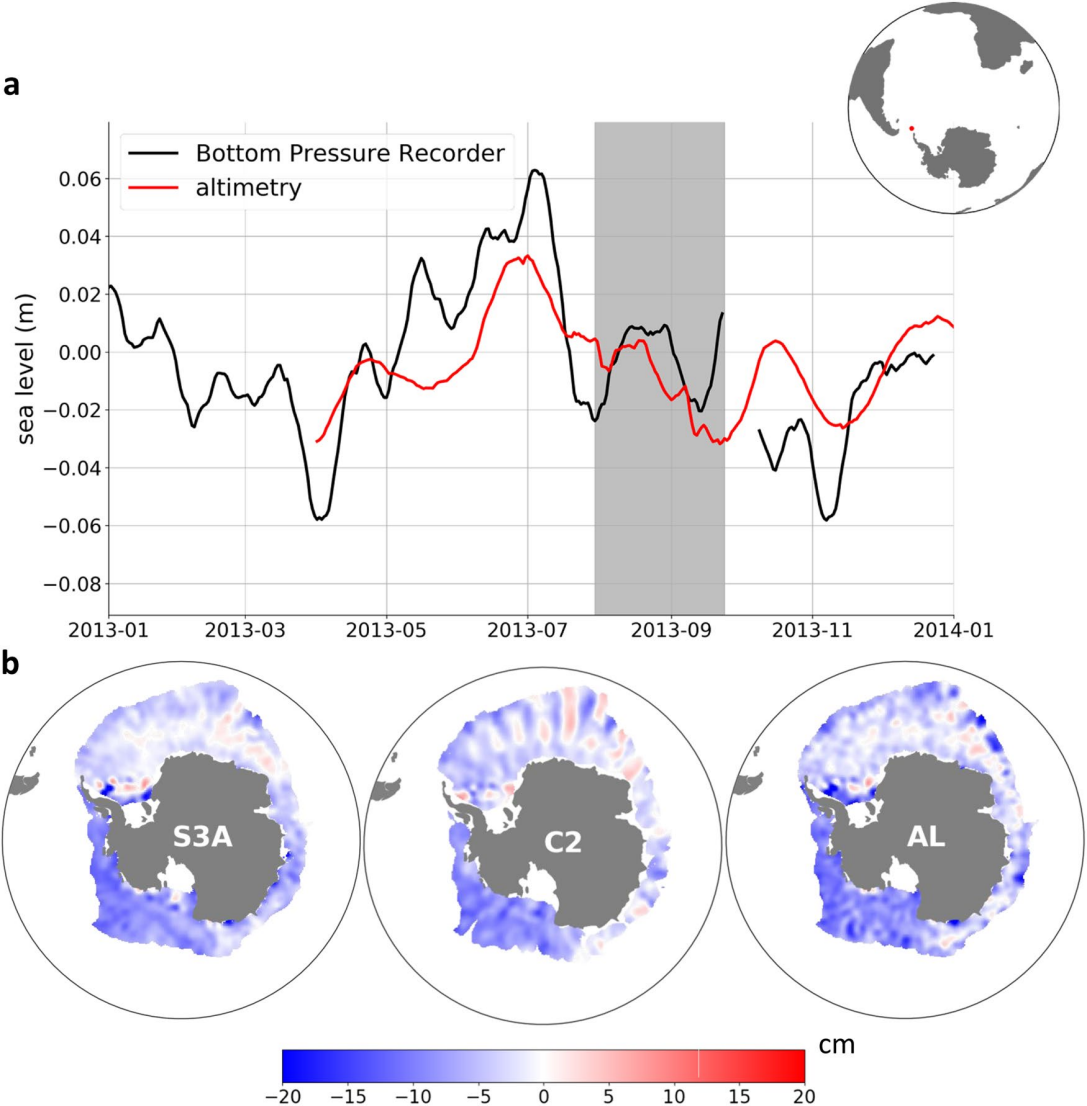
(**a**) Sea Level Anomaly (SLA) validation in the drake Passage. The lack line is the sea surface height from the Drake Passage Bottom Pressure Recorder, summed with the local dynamic height climatology to account for the steric height variations. The red line is the SLA from altimetry. Red dot on the map is the location of the Bottom Pressure Recorder (**b**) Sea Level Anomaly snapshots mapped from the three altimeters in the sea ice zone. Each SLA snapshot is mapped independently from each altimeter.

Bottom pressure observation obtained at 60.8°S, −54.7°E (https://www.psmsl.org/data/bottom_pressure/locations/1608.php) is converted in sea level anomaly and filtered with a 15-day running mean. This bottom pressure recorder covers 2012 to the end of 2013, so comparison with our product is only possible from March 2013 to December 2013. One issue of this comparison may be that the BPR only shows the variability related to the mass component of the SLA, and not the changes in the steric height. To have a better representation of the full SLA variability, we added to the BPR signal a monthly climatology of the dynamic height^54^ at the location of the BPR, linearly interpolated into a daily signal. BPR time series is compared with ~300 km filtered SLA product at the same grid point. Both time series are shown in Fig. 4a. The agreement between the two time series is good both during the ice-free and ice-covered seasons, with an overall significant correlation r = 0.65 over 232 days. Adding or not the dynamic height makes only few differences, as its full variability is only of the range of 2 centimeters. Without the dynamic height, the correlation is still significant but with a lower value of r = 0.61. We note that some variations differ within a month, and might depend on the altimetry sampling frequency at the location of the bottom pressure recorder. In particular, the correlation improves when averaging multiple grid points around the BPR, reaching 0.81 when averaging over a radius of 150 km around the BPR. In summary, the agreement with the bottom pressure recorder is good, but a longer and less sparse bottom pressure observation would be needed for a more extensive and statistically robust validation.

#### Consistency between altimeters in the sea ice regions

One alternative validation for our product is performed by comparing maps produced by the different individual altimeters. Although SARAL/AltiKa serves as a reference, since the other satellite records are corrected using monthly median offsets, the spatial patterns within the sea ice zone indicate local daily differences. It is also a way to evaluate the error induced by sampling differences and disparities within the various altimeter properties. All daily maps are filtered with a ~150 km Gaussian filter to filter out mesoscales which would be sampled differently by altimeter depending on their exact path and time of observation.

Concordance between the maps derived from each altimeter is estimated daily with the standard deviation of the height bias between the maps. From July 2016 to June 2018, the standard deviation ranges from 3 to 6 cm, with a median standard deviation of 4 cm for all altimeters, and a slightly better agreement (lower standard deviation) between C2 and S3A. A snapshot of SLA maps in the sea ice zones from each altimeter is shown Fig. 4b.

#### Error estimation from independent along-track measurements

**Fig. 5:**
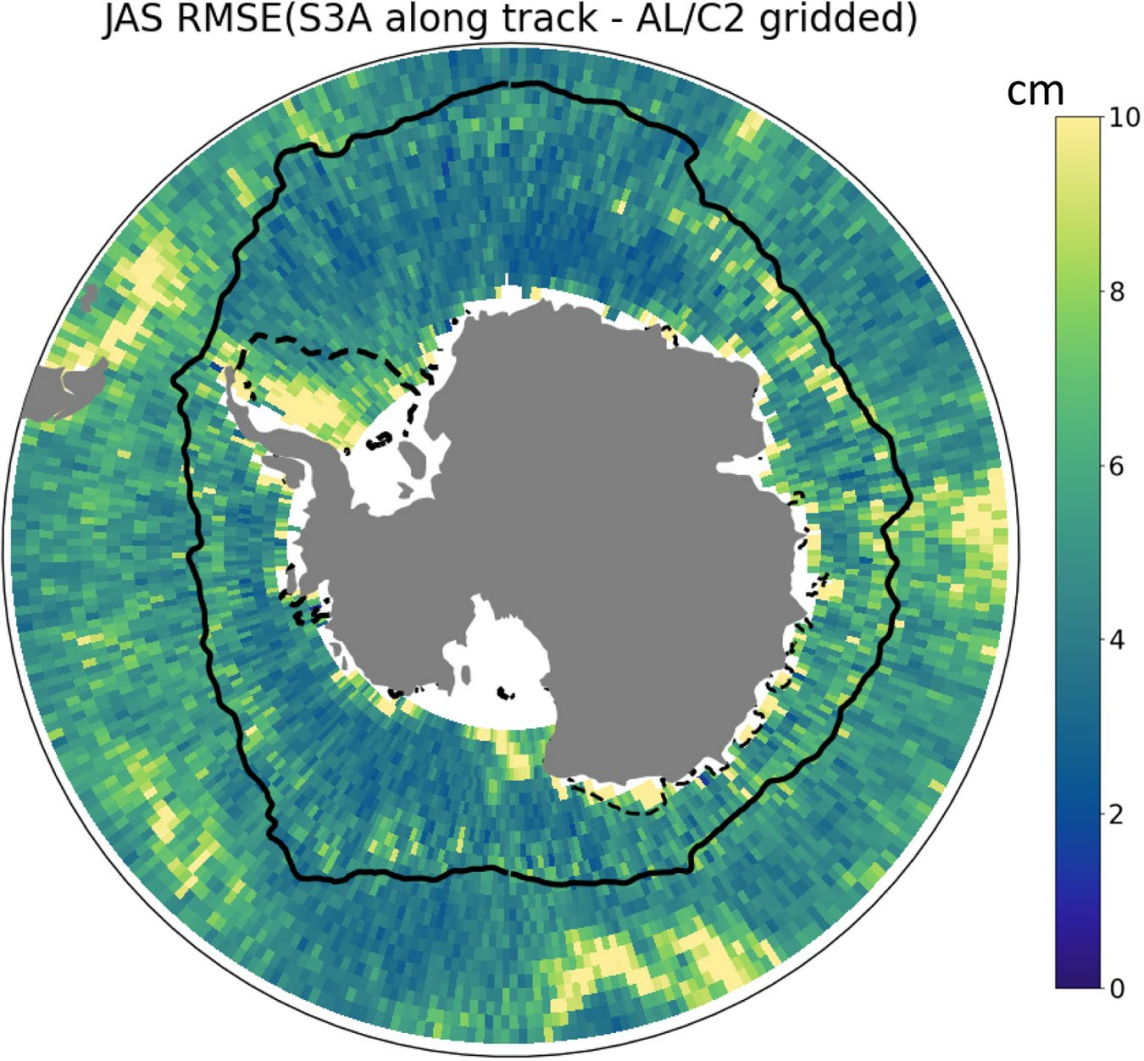
Standard deviation of the error between along track S3A (filtered with a 107-km running mean) and interpolated product constructed only with SARAL/AltiKa and Cryosat-2. This standard deviation was computed on the July-August-September months of 2016 and 2017. Black solid line is the mean 3% sea ice concentration contour for the July-August-September months of 2016 and 2017. Dotted line is the 3% contour of the minimum sea ice concentration over the years 2013-2019.

To evaluate the precision of the product in a 2-altimeter configuration, we compare the mapped product from SARAL/AltiKa and Cryosat-2 from July 2016 to July 2018 with the along-track SLA from Sentinel-3. The median zonal and meridional correlation scale south of 50°S is 107 km (Fig. 2a, b). Therefore, Sentinel-3 along-track data is filtered with a ~107 km running mean filter. The SARAL/AltiKa and Cryosat-2 dataset mapped on the 25km EASE2 grid is linearly interpolated on the tracks of Sentinel-3A measurements. Error is defined as $\left|SL{A}_{along\_track\_S3A}-SL{A}_{C2\_AL}\right|$. The root mean square error (RMSE) is computed each month on a 1x1° grid. Figure 5 shows the RMSE averaged over July, August, and September (JAS) from 2016 to 2017. RMSE values are different within various regions of the Southern Ocean. In the open ocean, the RMSE ranges from 4 to 10 cm in the most energetic jets of the ACC. In the sea ice zones, the RMSE is higher in the permanently ice-coverered regions of the Subpolar southern Ocean, reaching 10 cm regionally. In the seasonally ice-covered southern ocean, the standard deviation of the error ranges from 3 to 5 cm. Over 2 years, the median value of the RMSE in the permanently ice-covered Southern Ocean is 5.8 cm. In the seasonally ice-covered Southern Ocean and in the open ocean, the median RMSE are respectively 3.7 and 4.0 cm.

### Cryosat-2 induced pattern mitigation

**Fig. 6:**
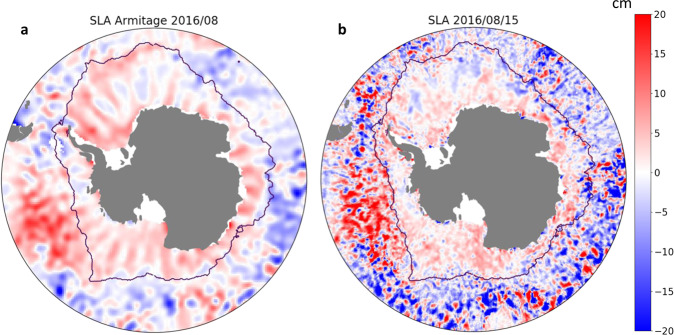
(**a**) Snapshot of Armitage SLA on 2016/09, showing a meridional pattern due to the orbit of Cryosat-2. (**b**) Snapshot of the Southern Ocean SLA product on 2016/09/15, showing a mitigation of this signal from the use of multiple altimeters.

Several SLA products for the subpolar Southern Ocean have been previously developed^20,21^. The observation-based product presented in this paper introduces several processing differences, as a long-wavelength error correction, the multimission combination, and among the other missions the use of a physical retracker for lead echoes for SARAL/AltiKa. Consequently, differences between our product and previous product are expected. The most notable difference when comparing monthly maps between our product and Armitage *et al*.’s product^20,21^ is that our product significantly reduces unphysical meridional stripes in SLA anomalies (Fig. 6). Previous products were based solely on Cryosat-2 observations, which orbit does not allow for an optimal temporal sampling over the ocean: neighboring regions are sampled with a time step of one month. Such a relatively long gap in time between the sampling of two neighboring regions, combined with the fact that SLA variability is large over one month, leads to such stripes when the product is interpolated and gridded (Fig. 6a). Our methodology that combines Cryosat-2 observations with observations from other satellites allows a strong mitigation of such source of error (Fig. 6b).

## Data Availability

The codes used to process the along track measurements and for the Optimal Interpolation (OI) are not available for public use as Collecte Localisation Satellite (CLS) and the Centre National des Etudes Spatiales (CNES) are the proprietary owners. However, these codes are extensively described in^55^ and^8^. The python code used for the comparison of the product with external sources of data are available at https://github.com/MatthisAuger/SO_SLA.
